# NUDGING FOR HEALTH: ON PUBLIC POLICY AND DESIGNING CHOICE ARCHITECTURE

**DOI:** 10.1093/medlaw/fwt022

**Published:** 2013-09-30

**Authors:** Muireann Quigley

**Affiliations:** * Centre for Ethics in Medicine, School of Social and Community Medicine, University of Bristol, UK

## Abstract

There have been recent policy moves aimed at encouraging individuals to lead healthier lives. The Cabinet Office has set up a ‘nudge unit’ with health as one of its priorities and behavioural approaches have started to be integrated into health-related domestic policy in a number of areas. Behavioural research has shown that that the way the environment is constructed can shape a person's choices within it. Thus, it is hoped that, by using insights from such research, people can be nudged towards making decisions which are better for their health. This article outlines how nudges can be conceived of as part of an expanding arsenal of health-affecting regulatory tools being used by the Government and addresses some concerns which have been expressed regarding behavioural research-driven regulation and policy. In particular, it makes the case that, regardless of new regulatory and policy strategies, we cannot escape the myriad of influences which surround us. As such, we can view our health-affecting decisions as already being in some sense shaped and constructed. Further, it argues we may in fact have reason to prefer sets of health-affecting options which have been intentionally *designed* by the state, rather than those that stem from other sources or result from *random* processes. Even so, in closing, this article draws attention to the largely unanswered questions about how behavioural research translates into policy and regulatory initiatives.

## I. INTRODUCTION

There have been recent moves in policy circles to embrace a host of strategies which are ostensibly aimed at encouraging and enabling individuals to lead healthier lives.^1^ These draw on behavioural research which purportedly shows that individuals are not the kind of rational, advantage-seeking decision-makers which some rational choice models predict.^2^ Such research, therefore, seeks social, cognitive, and other explanations of behaviour. Its main interest lies in what people actually do and why they do those things. In general terms, the conclusion reached is that people are lacking ‘clear, stable, or well-ordered preferences’ and that their behaviour is susceptible to the influence of ‘default rules, framing effects  …  and starting points’.^3^ It is hoped that, by using behavioural insights provided by such research, people can be ‘nudged’ towards making decisions which are better for their health.^4^ Strategies can involve ‘[c]hanging the way options are presented or altering the natural and physical environment [in order to] make it more likely that a particular choice becomes the natural or default preference’.^5^

Wherever institutions, private or public, can construct sets of options from which people choose, they have become ‘choice architects’.^6^ Thus, where the state pursues policies aimed at influencing the decisions that its citizens make regarding their health, it becomes a choice architect for its citizens' health. Such policies need not be confined to institutions whose main business is health (or health care). It might include initiatives which have health effects, even if they are based in other state run departments such as food, agriculture, or transport. For example, making bicycles available through public hire schemes may alter social norms and nudge people to cycle rather than taking other transport.^7^ In the UK, behavioural approaches (rightly or wrongly) have become an integral part of the public health strategy of successive Governments. The latest incarnation of this can be seen in its application to the policy arena of research findings from behavioural psychology and economics. To this end, the Cabinet Office has set up the Behavioural Insights Team (COBIT or the so-called nudge unit), which has made the public's health one of its priority areas.^8^

This article constitutes an analysis of these recent policy moves in respect of health with the aim of addressing some of the concerns which have been expressed regarding behavioural research-driven regulation and policy. Specifically, it examines the idea of influencing, shaping, and constructing choices through public policy. In order to do this, I look at the recent behavioural turn in law and policy, particularly the idea of ‘nudging’,^9^ and outline some of the research from the behavioural sciences which has caught the attention of government in the UK. I show how such moves reflect an underlying political ideology about the law and regulation and how this is already impacting on health-affecting regulation and public policy. Following this I argue that, although the new vogue in the policy arena involves influencing the choice architecture that surrounds us, many health-affecting choices are already in some sense ‘constructed’ or ‘shaped’. Given this, I look at whether we have any reasons, ethically-speaking, to prefer sets of health-affecting options which are *randomly* constructed as opposed to those which can be said to have been intentionally *designed* by the state. In so doing, I take issue with some of the arguments by commentators who are concerned about the effect of ‘government efforts to shape choices’ on our decision-making capacities.^10^ I contend that certain objections to state-mediated interventions imply that there is some sort of moral priority to be given to situations in which the choice architecture is due either to (a) random processes or (b) the result of the influence of private enterprise or interests rather than state-driven interests. I argue that no such moral priority ought to be accorded in this regard. Further, I argue that we might sometimes have reason to prefer a deliberately constructed health-affecting choice environment. Nonetheless, at the end of the article, I indicate that, given the number and complexity of influences on our health-affecting decision-making processes, there are largely unanswered questions about how behavioural research translates into policy and regulatory initiatives.

## II. NUDGING, CHOICE ARCHITECTURE, AND PUBLIC HEALTH POLICY

In liberal legal and political philosophy, autonomy and respect for the autonomous choices of individuals are often emphasised when examining how the law ought to operate and how social institutions ought to be structured.^11^ The law, along with the institutions and instruments through which it is operationalised, ought not be coercive or infringe excessively on a person's liberty and autonomy, or so the argument goes. A specific instance where such arguments are brought to bear is in the arena of public health and debates about what measures are acceptable to achieve desired public health outcomes (for example, decreasing smoking, alcohol intake, or obesity). Certain arguments in this arena either maintain that the law has no business interfering in a realm which is essentially about personal lifestyle choices^12^ or that, if health-affecting lifestyle decisions and activities do fall within the ambit of the law, the least intrusive or constraining approaches to regulating these ought to be used.^13^ Arguments along these lines could be seen as gaining some of their justificatory power from our traditional accounts of autonomy and rationality. Where individuals are seen as being rational actors then decisions about how to live their lives ought to be left up to them, (ostensibly) free from the long arm of the law. Thus, neoclassical accounts of rationality would support the argument that the law with respect to the public's health ought to be constructed in a manner which does not (unjustifiably) constrain the liberty of citizens. Likewise, the institutions and instruments through which the law operates, which have a bearing on the public's health, ought not to employ overly constraining methods. However, recently behavioural research is being used to challenge this position. We are, according to research in the behavioural sciences, imperfect decision-makers. We do not always act rationally in our own best interests. Instead, we are what Dan Ariely terms *predictably irrational*.^14^ Part of the reason for this is that we all fall foul of a number of cognitive biases which affect the way we make decisions. By taking such biases into account when formulating health policy, the Government hopes to be able to ‘nudge’ citizens to make better decisions in this respect.

A nudge (as opposed to some other intervention) is ‘an aspect of choice architecture that alters people's behaviour in a predictable way without forbidding any options or significantly changing their economic incentives.’^15^ Choice architecture simply refers to the context in which we choose and make decisions, but, as we will see below, this is important because the context itself can influence the way we think and the decisions we make. Thus, where our behaviour *changes* due to some aspect of the choice architecture which surrounds us we can be said to have been nudged. Richard Thaler and Cass Sunstein argue that nudges (properly conceived) belong to a political and economic ‘third way’ which they dub libertarian paternalism:^16^ ‘paternalism’ because they think ‘it is legitimate for private and public institutions to attempt to influence people's behaviour’ and ‘steer people's choices in directions that will improve the choosers’ own welfare'^17^ and ‘libertarian’ since they maintain that this ought to be done in a way that preserves ‘freedom of choice on grounds of either autonomy or welfare’.^18^ Take, for example, the potentially health-affecting nudge mentioned earlier in the introduction: cycle hire schemes in cities. The idea behind this is that ‘seeing more people cycle would create a new social norm and visual prompt, encouraging more people to want to cycle’.^19^ More bicycles in cities does not foreclose the option of taking the bus or driving, but does attempt to harness the powers of norms (of which more will be said below) to particular policy ends. In this case, there might be at least two such ends; first, to increase the amount of exercise that people take, and thereby contribute to making them healthier, by nudging them to cycle more and, second, to decrease the amount of congestion and pollution that results from cars and other vehicles. The economic incentives from the viewpoint of the cyclist do not necessarily change (something required by Thaler and Sunstein's own definition), since they need not hire the bicycles from the scheme as they may already have their own.^20^

The rhetorical appeal of the terminology used by Thaler and Sunstein is clear. In utilising the seemingly oxymoronic label, libertarian paternalism, they are indicating that they think such an approach to law and policy^21^ falls between the extremes of top-down command and control regulation on the one hand and laissez-faire libertarian market capitalism on the other.^22^ This policy turn in relation to public health is evident. In the short time, since COBIT was set up, behavioural approaches have started to be investigated and, to some extent, integrated into health-related domestic policy in a number of areas. Specifically, there has been a focus on organ donation, smoking, diet,^23^ alcohol intake, physical activity, and reducing prescription errors and missed appointments within the NHS.^24^ It is notable that the usual lifestyle factors (smoking, alcohol intake, exercise, and diet) are part of the latest policy moves. These are familiar targets in government public health strategies and not without good reason. Together poor diet, lack of exercise, smoking, and excessive alcohol intake represent a significant public health challenge. For example, in England 61.3% of adults are either overweight or obese^25^ and approximately 21% smoke.^26^ It is estimated that obesity costs the National Health Service (NHS) in excess of £5bn annually and is associated with type 2 diabetes, heart disease, and cancer, while smoking-related illnesses cost the NHS and society at large over £13.74bn annually.^27^ Part of the strategy in relation to alcohol is illustrative of the new approach and how the implementation of policy could alter choice architecture in way which may be health-affecting. In this regard, three of the major supermarkets have entered into a ‘responsibility deal’ with the Government and agreed not to display alcohol at the front of their stores.^28^ This initiative reflects the implementation of behavioural research, which shows that the way the environment is constructed can shape a person's choices within it. For example, in relation to food, it has been shown that ease of access, proximity to the food, and the amount of effort needed to be exerted to get it all affect consumption.^29^ Thus, it is hoped that by changing the choice architecture in the supermarket, that is changing the positioning of alcohol products, there will be a reduction in the amount being purchased.

As Yashar Saghai notes, however, we ought not to make the mistake of thinking that all nudges are forms of libertarian paternalism; the latter being a ‘justificatory strategy for a subset of nudges’.^30^ A key criterion of this subset of nudges is that they are for the benefit of those nudged, but not all nudges benefit the nudgee.^31^ This is evident from the Government's priority areas listed above. While strategies in relation to smoking, the salt content of food, and alcohol may seemingly be for the benefit of the nudgee, those in relation to organ donation, prescription errors, and missed appointments are not (at least in a direct sense). Take organ donation, for example. Since July 2011, those applying for or renewing their driving licence are required to answer one of three questions regarding registration on the NHS Organ Donor Register (yes, I would like to register, I do not wish to answer this question now, or I am already registered).^32^ Yet, while we might argue that we all benefit if more organs are available for transplantation, the nudgee in this case does not directly benefit. For the purpose of this article, my arguments can generally be taken to be referring to the subset of nudges which are intended to be for the benefit of those nudged. In addition, throughout this article, we should be careful not to elide two potential readings of what it means to be ‘nudged’ and which could be imputed from some of the literature referred to. If it is the case that a nudge is an aspect of the choice architecture which makes it more *likely* that a person's behaviour will be changed in a particular way then two possible analyses follow. On the one hand, we might regard a person as having been nudged regardless of the efficacy of the nudge employed; that is, whether or not it actually works (well). On the other, we might reserve the use of the term only for those cases where behaviour is in fact altered.^33^ For the purposes of my arguments, I use either ‘nudges’ or ‘nudge strategies’ to denote plans, policies, and attempts to nudge, whether successful or not. I apply active terms, such as ‘nudged’, for those situations in which behaviour change has occurred.

### A. (Public) Health and the Politics of New Regulatory Strategies

There have been calls for some time for a new approach to public health in the UK. This is consequent on the recognition and acceptance that health problems often have a complex and multi-faceted aetiology.^34^ In particular, there is evidence that a range of social and other factors influence individual health outcomes. These include early health status, educational attainment, employment (or lack thereof) and working conditions, and income level.^35^ This has in turn been acknowledged in a variety of Government reports.^36^ One consequence of problems which are multi-faceted is that any possible solutions may be equally difficult and thorny. As such, behavioural approaches represent just one part of the picture in relation to health. Nevertheless, although behaviour change approaches started under the previous Government and have been varyingly incorporated into policy by different state departments over the years, they have politically come to the fore under the current Government.^37^ Therefore, notwithstanding the wider health context, this article substantially focuses on nudging as the most recent policy incarnation of such approaches. The penetration of such strategies with regards to the regulation of health in this country seems to be a reflection both of an overarching political ideology and a drive towards minimally disruptive, market-preserving regulatory strategies which are seen as low-cost.^38^ With regards to the first of these, since 2010 the coalition Government's ‘Big Society’ agenda can be seen as containing the overarching themes which are the driving force behind many Whitehall initiatives. It is the manifestation of a political ideology which favours more decentralisation and de-regulation in its approach to governance than the previous incumbents.^39^ This move to decentralise in relation to health, for example, can be seen most clearly in the Health and Social Care Act 2012 which, after a turbulent passage through Parliament, gained Royal Assent last year. The Act, in abolishing NHS primary care trusts and strategic health authorities, makes local clinical commissioning groups the bedrock of the health service. Related to the political (ideological) commitments to light-touch regulation and government is a belief that changes in such a direction will be cost-saving. Thus, the enthusiastic adoption of nudging is at least partly down to ‘the promise of cost effectiveness; achieving “more for less”, particularly in public services’.^40^

Some policy commentators conceptualise nudging as an alternative to the law and regulation.^41^ This is understandable if we think of regulation in classical terms as being about ‘establishing, monitoring and enforcing legal rules’.^42^ Yet when purposively deployed by the state to achieve particular social policy ends, nudging could be seen as another regulatory technique in its arsenal. Roger Brownsword's broad definition takes regulation as ‘encompassing whatever measures regulators take to control and channel conduct in the desired way’.^43^ In this regard, nudge strategies can be seen as sitting within *design-based* approaches to regulation which, as Karen Yeung outlines, provide the state with tools which are not ‘the “traditional” policy instruments of command, competition, communication and consensus’.^44^ Regulation by design is often associated with what Brownsword terms ‘techno-regulation’.^45^ This is not about regulating technologies, but instead describes instances of where technology is used to ‘design-in a solution to a problem of which regulatees might not even be aware  …  and by-passes practical reason to eliminate all options other than the desired pattern of behaviour’.^46^ Examples of such regulation are computer software where the code can be written in such a way as to prevent unlicensed copying^47^ and designing cars in such a way that the engine will not start unless the seatbelt is fastened.^48^ Design-based regulation could be conceived of as almost synonymous with using technology as a regulatory modality.^49^ It can, however, also be construed in a much broader (and looser manner) than strictly technocratic fixes. Firstly, as the health-related examples in the next two sections will make clear, the design or alteration of the choice architecture which surrounds us can be used in order to try to achieve particular health policy goals. This need not be technology-driven. It encompasses a variety of means which includes not only the use of technology to influence citizens' decision-making, but also the physical design of places and spaces,^50^ and harnessing the power of different methods of information delivery. Secondly, unlike many techno-regulatory instruments which aim to inhibit particular actions and eliminate *all* choice options, a nudge does not (theoretically at least) foreclose options to those that are nudged. In general, nudges are being deployed in situations where individual choice is still thought to be important; this being exemplified by the focus on ‘lifestyle’ nudges by the government. This differs from examples such as the seatbelt, where a technocratic fix could be used as an effective means to enforce a pre-existing legal ban on driving without a seatbelt; legally, at least, there is no presumptive element of choice here. Contrariwise, there is apparent choice over other areas of our lives; for instance, what we eat.^51^ Nudges merely make it more likely that a person will choose in one direction rather than another.

In the sense that these new measures are aimed to bring about particular outcomes (by altering certain social norms and behaviours), yet ostensibly preserve choice, they can be seen as a form of (design-based) regulation-lite. They are part of the widening gamut of instruments that the government can (and do) call upon to regulate the health of its citizens. Although formal government-led behaviour change initiatives are in their infancy, with the nudge unit being barely three years old, such strategies seem set to have the political longevity. A recent report by one of the COBIT advisors recommends that the unit's remit be extended beyond its initial term.^52^ In addition, such strategies are (rightly or wrongly) viewed as a form of light-touch, low-cost regulation and, given the on-going economic pressures on state-run departments, are likely to still find favour under any successive regime, albeit in a potentially re-branded form. In order to better understand some of the potential issues and objections as well as benefits in this area, let us look briefly at how nudges are intended to work.

### B. Predicting Irrationalities

As noted already, recent research in the behavioural sciences purports to show that individuals do not always act rationally in their own best interests. One explanation for this is that we are prone to make certain predictable and systematic errors in judgement. We make such errors systematically not only in the sense that they are a regular occurrence in our decision-making processes, but because the biases which arise in the way we think can be notionally divided into system 1 and system 2 biases.^53^ These systems can alternatively be referred to as fast thinking and slow thinking.^54^ System 1 processes are automatic and largely non-voluntary, while system 2 thinking is slower, more deliberate, and more controlled.^55^ Biases and cognitive errors can arise due to ‘stumbles’ in either system.^56^ There are a number of heuristics (rules of thumb), biases, and effects which can all affect how we think.^57^ For instance, we categorise persons and objects based on how similar they are to stereotypes which we hold (representative heuristic).^58^ Doing so, however, can lead us to disregard other information which might have a bearing on what we are thinking about. Further, our assessment of the frequency or probability of an event is affected by the ease with which we can think of a pertinent or recent example of the occurrence (availability heuristic).^59^ We are also influenced by reference points and starting points. These can affect estimates that we make, including the value we place on things (anchoring, priming, and adjustment).^60^ Studies have also shown that we do not like losses and weigh them heavier than equivalent gains (loss aversion),^61^ when presented with options we tend to stick with the default one (status quo bias),^62^ and the manner in which information is presented to us affects our perception of that information (framing effects).^63^

The idea behind behaviour-driven health policy and law formation is that we can work either to eliminate or harness errors and biases^64^ in order to achieve certain health-affecting outcomes; for example, decreasing the amount of unhealthy foods that people eat and increasing the amount of physical activity they do. In this respect, the UK Government has produced a series of reports and policy documents which draw to varying extents on the existing body of behavioural research.^65^ Their MINDSPACE framework sets out what they see as ‘some of the most robust (non-coercive) influences on our behaviour’.^66^ The MINDSPACE acronym (Messenger, Incentives, Norms, Defaults, Salience, Priming, Affect, Commitment, and Ego)^67^ is intended to function as a checklist to be drawn on when making policy. This is set out in the table below,^68^ along with an indication of some of the heuristics, biases, and effects which are represented.

**Table:** 

Messenger (framing effect)	We are heavily influenced by who communicates information
Incentives (loss aversion and status quo bias)	Our responses to incentives are shaped by predictable mental shortcuts such as strongly avoiding losses
Norms	We are strongly influenced by what others do
Defaults (status quo bias)	We ‘go with the flow’ of pre-set options
Salience (framing effect)	Our attention is drawn to what is novel and seems relevant to us
Priming (anchoring heuristic)	Our acts are often influenced by sub-conscious cues
Affect	Our emotional associations can powerfully shape our actions
Commitment	We seek to be consistent with our public promises, and reciprocate acts
Ego	We act in ways that make us feel better about ourselves

There is not space here to discuss all of the factors which influence behaviour, but I want to briefly look at how some of our biases could be mitigated or harnessed in ways which are health-affecting. By referring to some proposed and potential policy or regulatory initiatives, which aim to influence us by changing the choice architecture, I illustrate how, in the context of health, we do not make decisions in a context-free environment. Choice architecture is all around us.^69^ As such, our choices are constantly at the behest of a myriad of influences. We do not, therefore, even need to consider *new* policy initiatives to see that our choice-environment and health-affecting decisions are to some extent already shaped and constructed.

### C. Constructing, Shaping, and Influencing (Health) Choices?

Let us look more closely at some strategies which are intended to alter particular target behaviour: increasing physical activity taken, decreasing the amount of alcohol consumed, and decreasing smoking. An overarching strategy in the behaviour change arsenal is to alter the norms around a particular activity or behaviour. One example already mentioned is the public cycle hire scheme. With such a scheme, an increased availability of bicycles could lead to an increase in the number of cyclists on the road. This would, hopefully, then nudge others into cycling by contributing to altering the norm around transport. In relation to alcohol, a campaign outlining how little people drink could be used to try to decrease the amount of alcohol being consumed. This would be in stark contrast to traditional public health campaigns which have tended to emphasise our excesses and their effects. However, people tend to overestimate what others drink.^70^ When we shift the emphasis of such campaigns, to highlight real (lower) levels of drinking, attention is called to what people actually do. Since we are influenced by what others do, a new descriptive norm is formed and this affects the social acceptability of the target activity. This approach works better than merely telling people what they ought to do since knowledge of what other people are actually doing or are perceived to be doing is more powerful.^71^ A similar norms-based strategy could be utilised to try and decrease the number of people who smoke.

While altering the norm might be a longer-term strategy, different influences can be used to contribute to this. For example, where public health campaigns convey information about the frequency of smoking or drinking an anchor can be created. Anchoring describes how we fasten on to particular ‘trait[s] or information when making decisions'.^72^ Therefore, presented with a lower frequency of smoking we may adjust our reference point and this, in conjunction with wanting to do what others do, could lead to lower numbers of smokers.^73^ Along with this, an approach discussed by the Government is to use the influence of making commitments; when these are made publicly or to significant others, individuals are more likely to follow through on their promises.^74^ As such, smokers wanting to quit could be encouraged to make a public commitment to friends or family regarding their stated goal. Other initiatives could attempt to harness the power of affect and salience.^75^ The driving idea in this regard is that altering our emotional states can change people's behaviour. Thus, for example, making physical activity fun and enjoyable could make it more likely that individuals would participate.^76^ Further, by making activities novel, they are made more engaging. This combined with targeting can make them seem more relevant to specific groups. Of course, the problem with novelty is that it wears off. Therefore, the long-term effects that might result from novel initiatives is an open question. While they could be used as a hook to kick start lifestyle changes, the longer term approach may lie in altering social and cultural norms surrounding a particular practice.

Where regulators and policy-makers take approaches such as those just outlined they are actively altering the context in which we make choices. From the examples given, we can see that this context can be influenced by presenting options in a different manner or by making changes to the environment in which decisions are made. These strategies demonstrate how the decisions and choices which we make can be *shaped* by a variety of factors around us, including the physical, environmental, social, and informational. This shaping of decisions is exactly what some commentators are concerned about (of which I will say more in the next section). However, ‘influence' does not simply appear *de novo* whenever a new policy is implemented. Prior to any new policy, individuals are already being influenced, albeit by a potentially different set of factors. Take, for example, a paradigmatic case of nudging: the cafeteria.^77^ Paul Rozin and colleagues have recently demonstrated that altering the layout of foods at a salad bar can have an effect on food consumption.^78^ They examined ‘the influence of minor changes in accessibility on food intake in a real world situation’.^79^ They found that intake of a particular food (e.g. broccoli or cheese) decreased when placed in a more inaccessible position and when serving tongs rather than spoons were used.^80^ Taking the findings of this study into account, should cafeterias implement a policy of placing healthier foods in more accessible locations on the salad bar? If they decide to do so, they are without doubt influencing (albeit in a potentially minor manner) the eating habits of their patrons. Yet, contrariwise, if they do *not* make any changes, and leave the salad bar as it normally is, they are still exerting an influence; they are, perhaps unbeknownst to themselves, nudging their customers to put the more accessible foods on their plates.

A similar point can be made with reference to default options. As commentators note, defaults are sticky.^81^ This is because when choosing we tend to exhibit preferences for the current state of affairs over some alternative.^82^ Given that this is the case, there is, therefore, something to be said for government, regulators, and policy-makers setting defaults in a manner that is beneficial to citizens. One interesting healthcare example, although not for the (direct) benefit of the person whose behaviour the default aims to influence, is that of mechanical ventilators in intensive care units. It has been demonstrated that changing the default settings on ventilators to ‘provide lower air volumes into patient's lungs' led to a 25% decrease in mortality.^83^ When the default setting was changed, this took advantage of the fact that the intensive care physicians (like all of us) exhibit a bias for the status quo. Yet before the default was altered the exact same bias was at play. Given this bias, we can imagine uses for defaults in the pursuit of improved public health outcomes; for instance, patients could be automatically opted-in to appointments as part of screening programmes, such as cervical smears for the early detection of cervical cancer, or for vaccinations as part of a vaccination programme.^84^ Individuals could always choose not to attend, but changing the default might make it more likely that they would turn up.

The lesson here about choice architecture is simple: we cannot escape the myriad of influences that surround us.^85^ They shape our decisions, our choices, and, cumulatively, our lives. In this regard, a plausible interpretation of the current state of health is that it is due to multiple factors and influences that have nudged us in the direction of obesity, diabetes, ill-health, etc. These nudges and other influences might include the decreasing availability of green spaces for play and exercise, the increasing availability of entertaining media which encourage non-movement, increased portion sizes of meals, the layout of our supermarkets and cafeterias, the lack of safe and accessible bicycle lanes, and many other factors. Each one of these occurrences on its own may not explain the overall health picture, but each has contributed to it and in unison may add up to give us a larger significant explanation. If this is correct, then there is a real sense in which our health-affecting decisions and choices have been shaped by those who *construct the contexts* in which these are made. In this regard, as noted in the introduction, we can think of public institutions as choice architects where they can construct or shape sets of options from which people choose and which are aimed at influencing the decisions made. If we accept the implications of behavioural research, it becomes clear that, regardless of the implementation of new health-affecting law and policy, we are already being nudged (if not always then often). The relevant issues, therefore, would not seem to be ones about choice architecture and choice construction *per se*, but ones about who the architect is and to what ends they are trying to exert influence. The pertinent question in this respect is whether we have reasons to prefer choice architecture that results from countless random influences or that which has been deliberately designed? And, with regard to the latter, whether there is any moral priority to be accorded to private and corporate actors who seek to influence us rather than to the state? In order to examine these questions let us look at some objections to behavioural approaches in general and nudging in particular.

## III. REGULATING HEALTH AND IMPLICATING (IR)RATIONALITY

The overarching tenor of concerns regarding nudges as (health) policy instruments revolves around the fact that changes, such as those described in the previous section, can affect decision-making and choices without the individual being aware that they have been nudged or influenced in this manner. The reason for this is that:

[T]he causal mechanism through which choice architecture is intended to work deliberately seeks to by-pass the individual's rational decision-making processes in order to channel behaviour in the direction preferred by the choice architect. The entire basis upon which such policies are constructed rests on the premise that, due to various cognitive ‘defects', individuals frequently fail to exercise their powers of reasoned self-deliberation, and this failure can be exploited to ‘nudge' choices in a particular direction.^86^

The fact that exploitation of our cognitive biases is central to successful behaviour change has prompted commentators to put forward several interrelated worries about nudge and behaviour-driven policy. First, nudges are neither libertarian nor paternalistic as is claimed.^87^ Secondly, to the extent that they aim to be liberty-preserving there ‘is a very thin understanding of liberty' at their core.^88^ Thirdly, they circumvent rational decision-making and could, therefore, be construed as antithetical to autonomy proper.^89^ Fourthly, in by-passing our rational processes, they may in the longer term lead to infantilisation and a decrease in individual responsibility.^90^ Finally, to the extent that the success of some nudges relies on individuals not being actively aware that they are being nudged, they involve deception^91^ and perhaps coercion.^92^ All of these are also tied to further questions about what the legitimate regulatory and policy ends of government ought to be; that is, is it a legitimate goal of government to nudge us towards health-affecting ends? There is not space in this article to deal with all of these matters, but in the discussion which follows I touch on some of them. I do this in order to illuminate, in due course, the arguments as they pertain to choice architecture which is intentionally *designed* by the state in order to attempt to regulate health-affecting behaviour.

### A. Liberty, Autonomy, and Control

Earlier, we saw that a nudge is ‘an aspect of choice architecture that alters people's behaviour in a predictable way without forbidding any options or significantly changing their economic incentives’.^93^ The supposed libertarian aspect of nudging lies in the fact that the particular nudge employed ought not to foreclose any options to the chooser; ‘it leaves the choice set essentially unchanged’.^94^ This is to be contrasted with more command-based approaches to regulating health mentioned earlier. Legislation which stipulates (wearing seatbelts) or proscribes (the smoking ban) certain courses of action purposefully forecloses options to the would-be chooser. It is part of the intended purpose of such laws and forms part of what some might find objectionable about outright bans. For this reason, one might welcome nudges to the regulatory arsenal. If they are libertarian as is claimed, and our option set does not change, what might be the problem? One answer is that the concern about nudging is *not* whether it is liberty-affecting in a narrow sense; that is, to do with altering or not the actual option-set from which we choose. Rather what is at issue are the implications for autonomy writ large.^95^ In this vein, Hausman and Welch argue that:

To the extent that they are attempts to undermine that individual's control over her own deliberation, as well as her ability to assess for herself her alternatives, they are prima facie as threatening to liberty, broadly understood [that is autonomy].^96^

Although (strict) nudges do not add or subtract from the options open to us, they do *alter the probability* that an individual will make one choice as opposed to another. For this reason, we might be concerned about how being nudged affects our judgements and decisions as purportedly autonomous persons.

The general organising idea of autonomy is that, in principle, individuals can be said to be autonomous to the extent that they govern or control their own lives.^97^ This notion is co-extensive with the view of autonomous individuals as self-determining.^98^ A particular liberal conception of autonomy is reflected in Isaiah Berlin's account of positive liberty. He says:

I wish my life and decisions to depend on myself, not on external forces of whatever kind. I wish to be the instrument of my own, not of other men's acts of will. I wish to be a subject, not an object; to be moved by reasons, by conscious purposes which are my own, not by causes which affect me, as it were, from outside. I wish to be somebody, not nobody; a doer – deciding, not being decided for, self-directed and not acted upon by external nature or by other men  …  ^99^

Although to varying degrees all law and regulation attempts to control or alter the behaviour of individuals or institutions,^100^ this account expresses what some find so objectionable about regulation and policy-making which sets out to alter our behaviour through means which potentially lack transparency and undermine individual control.^101^ We want our lives to be guided by our own hand, not by the hand of the state or Whitehall bureaucrats. We do not want (invisible) external forces working on us, guiding us to ends which may not be our own. We do not want just the gloss of superficial liberty. Instead, individual liberty is important to the extent that is related to how persons live their lives as autonomous beings. When Berlin poses the question ‘What, or who, is the source of control or interference that can determine someone to do, or be, this rather than that?',^102^ the answer for liberals is *the individual themselves*.^103^

It is for reasons related to this that Karen Yeung maintains that, in as much as nudges aim to be liberty-preserving, there ‘is a very thin understanding of liberty' at their core,^104^ while, relatedly, Luc Bovens argues that:

There is something less than fully autonomous about the patterns of decision-making that *Nudge* taps into. When we are subject to the mechanisms that are studied in ‘the science of choice', then we are not fully in control of our actions  …  these are cases of not letting my actions be guided by principles that I can underwrite. And in as much, these actions are non-autonomous.^105^

Yet we are subject to those mechanisms whether or not the state institutes policies which aim to influence our behaviour. This is because, as we saw in the previous section, we are constantly being acted upon by innumerable physical, social, environmental, and informational influences. In this respect, there is something equally non-autonomous about all manner of decision-making in our everyday lives. When I go to the supermarket to do my weekly grocery shop, a variety of factors might have a bearing on what I buy: what kind of mood I am in, whether I have eaten or skipped lunch, how the supermarket is laid out, the smells that pervade the bakery section, whether or not I bring a pre-prepared list of items I need, whether there are special offers on, and so on and so forth. To the extent that these contextual factors circumvent my more deliberate system 2 thinking, and impact on my automatic and unconscious system 1 processes, they have by-passed my rational judgement. This may involve a complete or a partial side-stepping of these processes. Decision-making in such contexts could be characterised as either (substantially) non-autonomous or merely less than fully autonomous. The former would encompass times when there is no engagement of the faculties that facilitate autonomy, while at other times the processes concerned may involve some automatic and some deliberative aspects. Either way, I cannot, therefore, be said to be (fully) autonomous with respect to those particular decisions. Thus, if the main concern is about the lack of autonomous decision-making simpliciter, then it has nothing to do with the involvement of government and policy-makers one way or the other.

In so far as there is reason to be worried about autonomy being subverted, it cannot be because non-autonomous choices and decision-making are antithetical to how our lives (as moral agents) go in general. It would not be practical, or indeed possible, to submit every choice, whim, preference, and decision to a protracted process of introspection and reflection. As David Archard notes ‘the value of autonomy is to be found in *the leading of lives*’.^106^ As such, we must get on with the business of leading them. Any conception or ideal of autonomy conceived of as set apart from the reality of contextual influences is a philosophical fiction. It is a fiction that needs to be abandoned and this means accepting our cognitive limitations and working with them. If we accept that autonomy need not be heroic^107^ and that our rationality is bounded by cognitive and other limitations,^108^ then we can see that not all non-autonomous decision-making need be considered undesirable. In fact, a certain level of non-autonomous functioning helps us go about our daily lives. This is not to say that it is morally desirable that all choices and decisions be non-autonomous, merely that it is perhaps the case that more critical reflective thinking is not always necessary (more on this will be said later). Nonetheless, even accepting that choice architecture is pervasive and impacts on our lives, there are questions to be asked about the kinds and sources of influences which ought to shape and construct our decisions and choices. It is to these I now turn.

### B. Random or Designed Choice Architecture?

One concern about nudging is specifically about ‘*government* efforts to shape choices'.^109^ For example, Hausmann and Welch claim that ‘[g]overnment action is subject to abuse'.^110^ Further they maintain that:

No matter how well intentioned government efforts to shape choices may be, one should be concerned about the risk that exploiting decision-making foibles will ultimately diminish people's autonomous decision-making capacities.^111^

Expressing a related concern, White argues that:

Rather than trying to ‘nudge' people into making certain choices, we should hold people responsible for the choices they do make—which does not guarantee they will always make the ‘right' choices, but they will be more likely to make choices that reflect their true interests without introducing moral hazard in the form of a system which enables mistakes.^112^

However, given that we cannot escape the nudge-laden contexts in which we make decisions, do we have any reason to prefer sets of health-affecting options which are *randomly* shaped or constructed as opposed to those which can be said to have been intentionally *designed* by the state? In asking this question, let me begin by clarifying what I mean by random as opposed to designed choices or option-sets. What I am referring to here is the manner in which the choice architecture surrounding us is formed. Our lived-environment, as we have already seen, consists of a multitude of different influences, physical, environmental, social, informational, and much more; each of these individually or in concert nudges us in different directions to diverse ends. This can be seen as randomly constructed to the extent that such influences have not been deliberately planned to bring about particular outcomes or ends. On the other hand, the choice architecture can be conceived of as (at least partially) designed where some agent or other actor intentionally devises^113^ and implements a course of action or intervention aimed at bringing about or altering a specific health-affecting behaviour.^114^

One reason for preferring a choice environment which has *not* been deliberately constructed (at the hand of any particular person or organisation) is because it might make it more likely, as White thinks, that we will ‘make choices that reflect [our] true interests'.^115^ One mistake in this respect would be to equate ‘random’ with ‘no effect' (which I am not suggesting that White himself makes). Countless influences on our lives and decisions might have haphazard and unintentional origins, but their non-deliberate nature does not imply that they have no (health) behaviour effects. The impact on behaviour might well be less marked than with purposive coordinated efforts, but some influence will still be exerted. Nonetheless, we might think that a choice environment which is the result of random non-guided processes gives autonomy a sporting chance in a way that deliberately constructed ones do not. Yet it would seem to be an open question whether or not choice architecture which is randomly generated aids or hinders us in making choices which reflect our true interests (whatever they may be).^116^ Where the numerous arbitrary forces that act on us somehow combine to nudge us in directions we would have chosen after reflective deliberation then they could serve to promote our true interests. Conversely, insofar as they converge to push us in other directions, they may well be detrimental to the realisation of these interests.

One response to the foregoing is to point out, as Yeung does, that what is at issue is the ‘*conscious and deliberate* attempt to shape the context in which people make decisions'.^117^ Amongst other concerns with the recent (health) policy moves, it is the overt deliberateness and explicitness of attempts by the state to alter its citizens' behaviour (by means which perhaps lack transparency) which troubles some. For example, Hausman and Welch contend that ‘an organized effort to shape choices  … appears to be a form of disrespectful social control’.^118^ Some regulatory initiatives can be thought of as being deliberate in the sense of having a particular aim or set of aims, as well as in terms of the way in which they are implemented and the tools chosen to do so. For example, the implementation of a piece of legislation (such as the smoking ban mentioned earlier) can be seen as the classical example of a deliberate form of regulatory action by the state (and is undeniably a form of ‘social control'). The various prescriptions or proscriptions contained in a piece of legislation undoubtedly form part of the choice architecture which surrounds us and, as is its purpose, alters or controls to a more or less successful extent the behaviour of its target institutions or individuals. However, not all choice architecture which could be attributed to the state can be construed as deliberately designed. Some elements in this regard may not be the result of any purposeful coordinated effort at influencing the decision-making of its citizens and, as such, could be understood as random (or if not random *per se*, cannot be seen as having just one aim or well demarcated set of aims). If we think about it in terms of policy-driven regulatory attempts, we can see that this is because a multitude of different factors might all have contributed to the drafting and implementation of a particular policy (or set of policies), the result of which means no singular purpose can be attributed to it; for example, the need for political compromise. Some recent forms of soft regulation, using tools such as nudges, may be seen as deliberate, yet with regard to systematic efforts to shape choices by drawing on the behavioural sciences, government, regulators, and policy-makers are relatively late to the party: ‘our social environment is already manipulated by the private sector to promote unhealthy choices for their own commercial ends’.^119^ The more focused and organised efforts of private industry and a variety of corporate bodies could be seen as more akin to intentional design (in this respect, it may be thought to be generally easier to discern the purpose of private industry, e.g., to maximise profits).^120^ An example touched upon in this paper already is the layout of supermarkets. Adrian Carter and Wayne Hall point out how the placement of different foodstuffs is used to encourage sales of particular items:

Everyday products such as milk and bread are placed at the back of the store so that customers must walk past more discretionary food items, increasing the chances of purchasing them, while items high in sugar and fat are located at the checkout to promote impulse buying.^121^

Another example of commercial interests at work is the tobacco industry which uses ‘role models' and branded packaging to try and increase the attractiveness of its products; ‘there is evidence suggesting that tobacco companies' efforts to associate themselves with positive images are effective in mitigating the impact of health warnings’.^122^ Thus, government and agents of the state, such as regulators and policy-makers, are not alone in trying to influence behaviour. Private industry and corporate actors are experts in this respect, but with detrimental effects on health:

[They] manipulate behaviour in ways that maximize the consumption of harmful products and increase the incidence of significant personal and social harm, such as obesity, hypertension, cancer, violence, and addiction.^123^

Given the pervasiveness of corporate influences, arguments that deny that it is legitimate for government to enter this arena are akin to according some sort of moral priority to the *status quo*; that is, to permitting private industry and corporate actors to have a relatively free reign in their attempts to influence our (health) behaviour. If we are worried about nudges and other behavioural influences, then we ought not to display ‘a selective concern about the ethics of governmental nudges'.^124^ In saying this, however, we should note that such selectivity is relatively easily addressed if one is not an extreme libertarian. One could deny that arguments against government policy aimed at altering its citizens' behaviour are tantamount to support for private industry to be permitted a free hand in the matter. Instead, one could argue that the current wrongs perpetuated by certain private sector organisations ought not to be added to by allowing policy-makers to do the same. But whatever we think about the government employing nudge policies themselves, they do not have to accord moral priority to corporate actors in this respect. It is not incumbent upon them to sit back in silence while private enterprise manipulates our preferences, choices, and decision-making. Rather the solution is to regulate the corporate sector more tightly and appropriately with respect to products which are health-affecting.^125^

Yet even if it is the case that tighter regulation is needed, this still leaves us with the problem of the effects of all manner of other random nudges and influences. As I argued earlier, it is an open question whether choice architecture which is random as opposed to designed can do any better at promoting our true preferences or interests (this and the relevant relationship to autonomy being what some are concerned about). If, however, nudge-type interventions are shown to be genuinely effective in the applied setting,^126^ this would give us reason to think that deliberately designed nudges could promote our interests as autonomous persons more effectively than a random assortment of influences. Nevertheless, because of the mechanism of action by which a nudge works, there are still reasons why they might be more ethically worrisome than instruments of command and control regulation such as outright bans. Integral to some objections regarding the introduction of tools such as nudges to our regulatory arsenal is that they seem to be aimed at controlling behaviour in realms which have traditionally been thought of as properly subject to individual choice (as demonstrated by the health arena where much of the focus is on influencing various ‘lifestyle' decisions). Some authors are concerned about the fact that (some) nudges work by by-passing our rational processes. One thing, however, that is apparent from what has been said in this article thus far is that there are many sorts of influences from a multitude of sources that by-pass our rational processes on a daily basis. In section 3A, I argued that a certain level of non-autonomous functioning is probably desirable. Nonetheless, a particular concern that has been raised in the literature which warrants some attention is the claim that, in the longer term, nudges may lead to infantilisation and a decrease in individual responsibility. We saw earlier, for example, that Hausman and Welch think that ‘exploiting decision-making foibles will ultimately diminish people's autonomous decision-making capacities’.^127^ While, in a similar vein, White argues that nudging relieves people of ‘responsibility for making these choices, and with the result that cognitive biases are accommodated rather than reduced or corrected’.^128^ Contrary to this, however, I suggest rather than leading to diminished decision-making capacities, as I am about to argue, it may in fact help to bolster them.

Studies suggest that individuals only have limited reserves of self-control and willpower (termed ego depletion); ‘if you have had to force yourself to do something, you are less willing or less able to exert self-control when the next challenge comes around’.^129^ A potentially health-affecting food-related example can be seen in an experiment where participants are given a mentally complex task to undertake. Here it was found that performance on the task was worse in those who earlier in the experiment had to resist eating more tempting foods such as chocolate and instead ate ‘virtuous foods such as radishes and celery'.^130^ According to Kahneman, the list of tasks known to be depleting includes ‘making a series of choices that involve conflict, trying to impress others, [and] responding kindly to a partner's bad behaviour’.^131^ The reason why this matters and is important for the argument at hand is that it has also been shown that depletion can have a range of effects on subsequent decision-making; for example, ‘deviating from one's diet, overspending on impulsive purchases, reacting aggressively to provocation,  …  [and] performing poorly in cognitive tasks and logical decision-making'.^132^ Further, linked to the idea of ego depletion is that of cognitive busyness: ‘[p]eople who are *cognitively busy* are  …  more likely to make selfish choices, use sexist language, and make superficial judgments in social situations’.^133^

If all of this is correct, then it may have substantive implications for persons as they go about their lives. Often we lead hectic and tiring lives and when our cognitive capacities have been depleted, or our efforts are being taken up with a multitude of tasks, we are not going to be operating at our optimal decision-making capacity. Consequently, it is plausible to think that our cognitive reserves ought not to be exhausted simply in the everyday struggle to cope with the vagaries of life. If this is the case, then some decisions must be made about where our efforts are best directed. If particular classes of decisions and choices were made easier to get ‘right', this would free up our decision-making faculties for other perhaps more important decisions. For example, when we approach the food bar in our workplace cafeteria, as we have already seen, its layout may nudge us towards certain food choices. Where the food is laid out in a manner that nudges us towards the healthier food options, it is conceivable that we would exert less cognitive effort in resisting the not so healthy ones. If this is correct, then our cognitive reserves are preserved for other (classes of) decisions we might need to make in the course of our day. Generalising this to action at state level, if we think it is a legitimate goal of government to safeguard, and indeed promote, the health of its citizens, the use of health-affecting nudges could help not only to achieve the desired health ends, but give citizens more cognitive space in general. There is, after all, something to be said for ‘freeing individuals from other irrelevant influences'.^134^ The consequence of this would be that, far from actually leading to infantilisation, certain well-judged nudges or other applications of behavioural research could actually aid us, with the right environmental support, in becoming more sophisticated and autonomous moral thinkers and actors.^135^ This argument does, of course, mean that we need to decide which categories of decisions and choices it is legitimate for the state to influence. The difficulty this then raises is whether the ends and interests of the nudger and the nudgee are appropriately aligned. There is a line to be drawn between what ought and ought not to be out of bounds (in terms of the regulation of the health of citizens) for would-be governmental nudgers. Nonetheless, it does at least suggest that nudge policy need not necessarily lead to a diminishment of individual responsibility or autonomy in decision-making.

## IV. CONCLUDING REMARKS: NUDGES AS HEALTH POLICY INSTRUMENTS?

In this article, I have outlined recent health policy moves which aim to encourage and enable individuals to lead healthier lives and argued that we can situate nudges within the set of strategies encompassed by design-based regulation. While it is, of course, not self-evident that what is better for health (whatever that might mean) is always better for particular people, ‘public policy inevitably should and does attempt to objectively evaluate better life choices'.^136^ Several commentators have expressed concerns about behavioural-driven regulation and policy; above all, they are concerned with the deliberate alteration and construction of our choice environments. This article has made it clear that we cannot escape choice architecture and, for that reason, we can view our health-affecting decisions as already being in some sense shaped and constructed. Further, it is not obvious that deliberately designed choice architecture is hostile either to our true interests and ends or to leading lives as autonomous persons. However, even if this is the case, one might respond that when it comes to designing and implementing public policy there is an epistemic difficulty. As White notes:

[R]egulators can never know what an agent's true interests are without observing her free choice or obtaining explicit consent. Instead, designers of choice situations or default choices substitute their own estimation of the interests of those they purport to regulate.^137^

This may be correct, but even if government does not get our preferences and interests exactly right, health-affecting nudges are at least an attempt to guide us towards *potentially* justifiable ends, such as better health, well-being, and happiness.^138^ A thorough-going analysis of how far they ought to go in this respect and the exact scope of legitimate state interference is a task for another time. However, here I have indicated that, whatever the legitimate ends of government consist of, they do not (and in practice cannot) entail that the state need remain silent or agnostic on matters affecting the health of its citizens, especially while private industry and corporate actors carry on regardless. Further, it is entirely plausible to think that influencing certain sets of decisions made by citizens falls within the remit of government. Robert Frank maintains that:

Historically, the main mission of government has been not to prevent people from making stupid decisions but to resolve collective action problems: to provide public goods such as roads, schools, and national defense; to provide a social safety net; to protect the environment; and so on.^139^

There are, however, at least two related situations in which preventing people from making ‘stupid decisions' could be seen as a legitimate sphere of action for government and regulators; first, where doing so prevents harm to others and, secondly, where doing so actually helps to resolve the problems of collective action. Often health-affecting choices do not merely have effects for the individual concerned.^140^ Take for example unhealthy eating habits; say a person with a continuous diet high in refined sugar and saturated fats. These may impact on those around them; their family and friends if they become ill and, more importantly in the context of the discussion here, the state in terms of the cost of health care or work days lost. The acceptability of state action in this case may stem from the legitimacy of preventing higher health care and other costs where these would mean fewer available resources for other citizens. Secondly, this suggests that people's choices are part of the collective action problem. Where the decisions and choices that individual citizens make mean that there are fewer resources in the state pot, this will have an impact on the state's ability to effectively pursue other goals, such as the provision of education, safe roads, further health care, etc. Therefore, from a pragmatic perspective, ‘if government is to deliver services such as health care and the like, it would be desirable to avoid the waste wrought by cognitive errors’.^141^ Even so, whether or not interventions such as nudges, which aim to influence the behaviour of individuals, are appropriate (and indeed efficacious) will depend on the specifics of the proposals at hand.

As Adam Burgess remarks, ‘the choice [for government] is often between *different forms* rather than between regulating or not’.^142^ In this respect, we might agree with Russell Korobkin that ‘the battle to separate the economic analysis of legal rules and institutions from the straightjacket of strict rational choice assumptions has been won’.^143^ Yet, it remains to be seen whether nudging will be effective as a health policy tool. As such, there is a pragmatic empirical concern about nudges; put simply they might not work. First, much of the behavioural research that has been done, and that policy-makers are attempting to draw on, has been done in controlled laboratory(-like) settings.^144^ Thus, the lack of empirical evidence from the applied setting is a potential reason to doubt the efficacy of behaviour change interventions. Secondly, some nudges may work better than others; for example, Amir and Lobel speculate that biases which arise due to our automatic system 1 processes may be easier to correct than ones that emanate from our more deliberate system 2 ones.^145^ Thirdly, even if certain nudges and other interventions do work, they may not be enough on their own to bring about the desired change or to ‘solve complex policy problems'.^146^ As I noted in section 2 of this article, it is probable that a range of social, economic, and other factors influence a person's health. Behavioural approaches by themselves are not a panacea. Where a substantial cause of the target health problem is due, for example, to some economic or social disadvantage then a nudge may not be enough. More thorough-going and interventionist measures may be required along with policies which can bring about the requisite social change.^147^ Where policy-makers attempt to do this, we must guard against what has been labelled ‘lifestyle drift'. This is the ‘tendency for policy initiatives on tackling health inequalities to start off with a broad recognition of the need to take action on the wider social determinants of health (upstream), but which, in the course of implementation, drift downstream to focus largely on individual lifestyle factors’.^148^ Given, as outlined at the beginning of this article, that the substantive focus of Government attention regarding health-affecting nudges is on the traditional lifestyle-focused health culprits (smoking, diet, alcohol, etc), there is a danger that the opportunity to take a wider systems approach is lost.^149^ Finally, there may be as yet unknown unintended consequences which flow from implementing behaviour change policies.^150^ There is, therefore, a largely unanswered question about how such research, given the complexity of the lived environment and the myriad of influences on our health-affecting decision-making processes, translates into policy and regulatory initiatives. Yet, despite all of these concerns, this does not mean we ought to abandon behavioural approaches to regulating health. Instead, government ought to ensure that pilot studies are properly carried out before any wholesale policy changes are brought in.^151^ The state can already be viewed as being a choice architect for its citizens' health. The challenge that is faced, therefore, by its regulators and policy-makers is to exert their influence in a manner which is not only legitimate, but also empirically robust.

## Footnotes

1 See, for example, HM Government, *Healthy Lives, Healthy People: Our Strategy for Public Health in England* (The Stationary Office 2010). Available at <http://www.dh.gov.uk/health/2011/07/healthy-lives-healthy-people/> last accessed 2 July 2013.

2 For example, G Becker, ‘Irrational Behavior and Economic Theory’ (1962) 70 J Pol Econ 1; G Becker, *The Economic Approach to Human Behavior* (University of Chicago Press, Chicago 1978, 1990).

3 C Sunstein and RH Thaler, ‘Libertarian Paternalism is Not an Oxymoron’ (2003) 70 U Chic L Rev 1159, 1161.

4 It should be noted that so-called nudging is not limited to health, but can encompass all sorts of social policy goals. The work of Richard Thaler has been particularly influential in bringing this approach to the table in UK policy circles. See Sunstein and Thaler, above, n 3, RH Thaler and C Sunstein, ‘Libertarian Paternalism’ (2003) 93 Am Econ Rev 175; RH Thaler and C Sunstein, *Nudge: Improving Decisions about Health, Wealth, and Happiness* (Penguin Books, London 2008, 2009).

5 House of Lords Science and Technology Select Committee, *Behaviour Change* (The Stationery Office, London 2011) 11.

6 Thaler and Sunstein 2009, above, n 4, ch 5.

7 Cabinet Office Behavioural Insights Team. Applying Behavioural Insight to Health 2010, 22. Available at <http://www.cabinetoffice.gov.uk/sites/default/files/resources/403936_BehaviouralInsight_acc.pdf>.

8 See, for example, Cabinet Office Behavioural Insights Team, *Applying Behavioural Insight to Health* 2010 (available at <http://www.cabinetoffice.gov.uk/sites/default/files/resources/403936_BehaviouralInsight_acc.pdf> last accessed 2 July 2013) and HM Government, above, n 1. Additionally, this approach is gaining ground in France in relation to health initiatives see Centre for Strategic Analysis, *Improving Public Health Prevention with Behavioural, Cognitive & Neuroscience* 2010 (available at <http://oullier.free.fr/files/2010_Oullier-Sauneron_CAS-Neuroscience-Prevention-Public-Health_Book_Neuroeconomics-Behavioral-Economics-Neuromarketing.pdf> last accessed 2 July 2013).

9 Much of what is said here is also applicable to influences which would not be considered as nudges *per se*. However, we should guard against conflating ‘nudge’ with ‘influence’, a slippage which seems to have occurred as the idea of nudging has permeated popular consciousness and been adopted as a policy approach. For an analysis which differentiates nudges from other categories of influence see Y Saghai, ‘Salvaging the Concept of Nudge’ (2013) 39 J Med Ethics 487.

10 DM Hausman and B Welch, ‘Debate: To Nudge or Not to Nudge’ (2010) 18 J Pol Phil 123, 135. Similar concerns have been raised, among others, by MD White, ‘We've Been Nudged: The Effects of the Downturn on Dignity and Responsibility’ in MA Starr (ed), *Consequences of Economic Downturn: Beyond the Usual Economics* (Palgrave MacMillan, New York 2011) 103; L Bovens, ‘The Ethics of Nudge’ in T Grüne-Yanoff and SO Hansson (eds), *Preference Change: Approaches from Philosophy, Economics and Psychology* (Springer Dordrecht, London 2009) 207; E Selinger and KP Whyte, ‘Competence & Trust in Choice Architecture’ (2010) 23 Know Techn Pol 461.

11 See, for example, J Feinberg, *The Moral Limits of the Criminal Law: Harm to Self* (Oxford University Press, Oxford 1986), chs 17 and 18.

12 For example, R Epstein, ‘In Defense of the Old Public Health: The Legal Framework for the Regulation of Public Health’ (2004) 69 Brook L Rev 1421. For a critique of Epstein's and other libertarian approaches to public health see J Coggon, *What Makes Health Public? A Critical Evaluation of Moral, Legal, & Political Claims in Public Health* (Cambridge University Press, Cambridge 2012) 153–63.

13 See, for example, R Upshur, ‘Principles for the Justification of Public Health Intervention’ (2002) 93 Can J Public Health 101, 102. See also JF Childress, RR Faden and others, ‘Public Health Ethics: Mapping the Terrain’ (2002) 30 J Law Med Ethics 170, 173. However, the claim (often taken as self-evident) that the least restrictive means is the (legally and) morally desirable course of action requires more work. The latter paper does suggest the least infringement principle as a means of resolving conflicts amongst competing moral considerations and outlines the need to take into account other factors such as effectiveness and proportionality.

14 D Ariely, *Predictably Irrational: The Hidden Forces that Shape Our Decisions* (Harper, London 2009).

15 Thaler and Sunstein 2009, above, n 4 at 6.

16 Ibid at 253–4. For a critique of *Nudge* as an ambitious political project see P Schlag, ‘Nudge, Choice Architecture, & Libertarian Paternalism’ (2010) 108 Mich Law Rev 913.

17 Sunstein and Thaler 2009, above, n 3 at 1162.

18 Ibid at 1160.

19 COBIT, above, n 7 at 22.

20 In any case even if a person was initially nudged to buy a bicycle, the cost may even out over time if they save money on fuel or on the cost of public transport.

21 We should be careful to distinguish between the idea of using the behavioural sciences to inform law and policy and the claim that any strategies adopted ought to be justified by an appeal to libertarian paternalism as the underlying political philosophy. In this article, I do not address whether libertarian paternalism is legitimate (or indeed preferable to other approaches to law and policy) in this respect, but instead focus on the issues relating to altering the choice environment in which citizens make health-affecting decisions.

22 See A Burgess, ‘Nudging Healthy Lifestyles: The UK Experiments with the Behavioural Alternative to Regulation and the Market’ (2012) 1 EJRR 3. As evidence that such strategies could indeed be perceived as a real third way, government and policy-makers on both sides of the Atlantic (Obama's Democrats and Cameron's Conservatives) have taken up the libertarian paternalist agenda with enthusiasm. In the USA, its influence is most evident with the appointment of Cass Sunstein as the Administrator of the Office of Information and Regulatory Affairs (although he stepped down from this post in 2012). In the UK Richard Thaler acts as an advisor to the Cameron Government.

23 In particular, in 2010/11, there was a focus on the salt content of food. See Cabinet Office Behavioural Insights Team, *Behavioural Insights Team Annual Update 2010–11*, 7–9 (available at <http://www.cabinetoffice.gov.uk/sites/default/files/resources/Behaviour-Change-Insight-Team-Annual-Update_acc.pdf> last accessed 2 July 2013) and *Salt Strategy Beyond* 2012 (available at <https://responsibilitydeal.dh.gov.uk/wp-content/uploads/2013/03/Salt-Strategy-Beyond-2012.pdf> last accessed 2 July 2013). Although a recent report by the Institute of Medicine demonstrates that the evidence on sodium intake is not as clear cut as often assumed. See Institute of Medicine, *Sodium Intake in Populations: Assessment of Evidence* (National Academies Press, Washington 2013) (available at <http://www.nap.edu/catalog.php?record_id=18311> last accessed 2 July 2013).

24 See *Behavioural Insights Team Annual Update 2010–11*, ibid and Cabinet Office Behavioural Insights Team, *Behavioural Insights Team Annual Update 2011–12* (available at <https://www.gov.uk/government/uploads/system/uploads/attachment_data/file/83719/Behavioural-Insights-Team-Annual-Update-2011-12_0.pdf> last accessed 2 July 2013).

25 Department of Health, *Healthy Lives, Healthy People: Call to Action on Obesity*, 2011, 10 (available at <https://www.gov.uk/government/uploads/system/uploads/attachment_data/file/134840/dh_130487.pdf.pdf> last accessed 2 July 2013).

26 Department of Health, *Healthy Lives, Healthy People: Tobacco Control Plan*, 2011, 15 (available at <https://www.gov.uk/government/uploads/system/uploads/attachment_data/file/135349/dh_124960.pdf.pdf> last accessed 2 July 2013).

27 See, respectively, DoH, above, n 25, 16 and ibid, 17.

28 COBIT, above, n 23 and Department of Health, *The Public Health Responsibility Deal* (Crown Copyright 2011) 24.

29 See, for example, P Rozin and others, ‘Nudge to Nobesity I: Minor Changes in Accessibility Decrease Food Intake’ (2011) 6 Judgm Decis Mak 323.

30 Saghai, above, n 9, 2.

31 Ibid.

32 See <http://www.nhsbt.nhs.uk/about/partners_and_supporters/dvla.html> last accessed 2 July 2013.

33 Thank you to one of the anonymous reviewers for this point.

34 See, for example, Independent Inquiry into Inequalities in Health, *Report of the Independent Inquiry into Inequalities in Health* (The Stationery Office, London 1998); The Marmot Review, *Fair Society, Healthy Lives: Strategic Review of Health Inequalities in England post-2010* (available at <http://www.instituteofhealthequity.org/projects/fair-society-healthy-lives-the-marmot-review> last accessed 2 July 2013).

35 Marmot Review, ibid, ch 2. Although note that there is concern in some quarters regarding the causal pathways that may (or may not) underlie such findings. For example, Gopal Sreenivasan maintains that ‘Each distinct social variable might function as a “marker” for a different underlying causal factor, different social variables might function instead as alternative markers for the same underlying causal factor, or there may be some mixture of both.’ See ‘Justice, Inequality, and Health’ in EN Zalta (ed), *The Stanford Encyclopedia of Philosophy* (Spring 2009 Edition) (available at <http://plato.stanford.edu/archives/spr2009/entries/justice-inequality-health> last accessed 2 July 2013).

36 For example, Government Office for Science, *Tackling Obesities: Future Choices – Project Report* (2nd edn, 2010); see, in particular, the obesity systems map on p. 85 (available at <http://www.bis.gov.uk/assets/foresight/docs/obesity/17.pdf> last accessed 2 July 2013). See also Department of Health, *Tackling Health Inequalities: 10 Years On* (Crown Copyright 2009) and *Healthy Lives, Healthy People*, above, n 1, 26. This has tackling the social determinants of health as one of its aims.

37 P John and L Richardson, *Nudging Towards Localism* (British Academy, London 2012) 18–25.

38 See HM Government, *One-in, One-out: Third Statement of New Regulation* (Department for Business, Innovation and Skills, London 2012). For examples of the move in this direction in relation to general regulations across sectors, as well as health and social care see the Cabinet Office's ‘Red Tape Challenge’: <http://www.redtapechallenge.cabinetoffice.gov.uk/home/index/> last accessed 2 July 2013. Although a move in this direction was also evident under the previous Government. For example, a report by the Better Regulation Commission stated that ‘[a]lternatives to classic regulation will often work better, stimulate more enterprise and innovation, promote greater freedom and choice, inspire greater self-reliance and responsibility, be cheaper and impose fewer burdens.’ See *Risk, Responsibility, Regulation: Whose Risk is it Anyway?* (BRC, London 2006) 37 (available at <http://webarchive.nationalarchives.gov.uk/20100402230200/http://archive.cabinetoffice.gov.uk/brc/upload/assets/www.brc.gov.uk/risk_res_reg.pdf> last accessed 2 July 2013).

39 Burgess, above, n 22, 7. For a brief overview of decentralisation under the current Government, see HM Governement, ibid, ch 5.

40 Ibid.

41 See, for example, P Rainford and J Tinkler, ‘Designing for Nudge Effects: How Behaviour Management Can Ease Public Sector Problems’ in *Innovating Through Design in Public Sector Services Seminar Series* (LSE Public Policy Group 23 February 2011) 3 (available at <http://eprints.lse.ac.uk/37810> last accessed 2 July 2013).

42 K Yeung and B Morgan, *An Introduction to Law and Regulation* (Cambridge University Press, Cambridge 2007) 3.

43 R Brownsword, *Rights, Regulation, and the Technological Revolution* (Oxford University Press, Oxford 2008) 7. For an article on the different understandings of regulation see J Black, ‘Critical Reflections on Regulation’ (2002) 27 Aus J Leg Phil 1.

44 K Yeung, ‘Towards an Understanding of Regulation by Design’ in R Brownsword and K Yeung (eds), *Regulating Technologies* (Hart Publishing, Oxford 2009) 79, 80.

45 R Brownsword, ‘Code, Control, and Choice: Why East is East and West is West’ (2005) 25 LS 1, 3.

46 Ibid at 4. Brownsword does not wholeheartedly endorse or reject techno-regulatory tools in this article, but instead draws our attention to the tension between control and choice which a move to technocratic solutions to social problems presents. See also R Brownsword, ‘What the World Needs Now: Techno-Regulation, Human Rights and Human Dignity’ in R Brownsword (ed), *Human Rights* (Hart Publishing, Oxford 2004) 203, 230. Here Brownsword seems to envisage techno-regulation in a much less stringent manner as merely encompassing the use of technology to aid regulation, although a central worry is about a move to techno-regulatory tools that leaves no room for choice.

47 L Lessig, ‘The Zones of Cyberspace’ (1996) 48 Stanford Law Rev 1403, 1408. See also ibid at 3 and Yeung and Morgan, above, n 42, 102–5.

48 K Yeung, ‘Can We Employ Design-Based Regulation While Avoiding *Brave New World*?’ (2011) 3 Law Innov Tech 1, 3.

49 K Yeung and M Dixon-Woods, ‘Design-based Regulation and Patient Safety: A Regulatory Studies Perspective’ (2010) 71 Soc Sci Med 502, 503.

50 Yeung, above, n 44, 81–2.

51 Although we will see a little later why this may only be ‘apparent’ choice.

52 John and Richardson, above, n 37, 7–8.

53 KE Stanovich and RF West, ‘Individual Differences in Reasoning: Implications for the Rationality Debate?’ (2000) 23 Behav Brain Sci 645, 658.

54 For an engaging overview of this see Daniel Kahneman's latest book, especially part 1, which derives its title from this distinction: *Thinking, Fast and Slow* (Allen Lane, London 2011). Kahneman won the Nobel Prize in Economics in 2002 (although he is a psychologist) for his work with the late Amos Tversky on judgement and decision-making. He is generally credited with being the father of behavioural economics.

55 Ibid at 20–1.

56 O Amir and O Lobel, ‘Stumble, Predict, Nudge: How Behavioral Economics Informs Law and Policy’ (2008) 108 Columbia Law Rev 2098, 2110. I take the reference to ‘stumbles’ from this article where the authors open by noting that ‘[t]o stumble is human’.

57 For an entertaining overview see Thaler and Sunstein 2009, above, n 4, 24–40. These have been studied by psychologists over the years building on the seminal work of Amos Tversky and Daniel Kahneman who conducted studies of how we think under conditions of uncertainty. See A Tversky and D Kahneman, ‘Judgement Under Uncertainty: Heuristics and Biases’ (1974) 185 Science 1124.

58 Tversky and Kahneman, ibid, 1124–7.

59 Ibid at 1127–8.

60 Ibid at 1128–30.

61 D Kahneman, JL Knetsch, and RH Thaler, ‘Anomalies: The Endowment Effect, Loss Aversion, and Status Quo Bias’ (1991) 5 J Econ Perspect 193, 199–203.

62 Ibid at197–9.

63 A Tversky and D Kahneman, ‘The Framing of Decisions and the Psychology of Choice’ (1981) 211 Science 453.

64 Amir and Lobel, above, n 56, 2114 and 2117.

65 See COBIT, above, n 7; COBIT, above, n 23; Department of Health, *The Public Health Responsibility Deal* (Crown Copyright 2011); HM Government, above, n 1; P Dolan and others, *Mindspace: Influencing Behaviour Through Public Policy* (Institute for Government 2010) (available at <http://www.instituteforgovernment.org.uk/images/files/MINDSPACE-full.pdf> last accessed 2 July 2013).

66 Ibid at 8. Note that this report is not an official Government policy document rather it is a discussion document. However, the framework has been incorporated into subsequent policy documents, see COBIT, above, n 7.

67 This would seem to be a (longer) version of Thaler and Sunstein's own acronym ‘NUDGES’: iNcentives, Understand mappings, Defaults, Give feedback, Expect error, and Structure complex choices (above, n 4, 109).

68 Dolan and others, above, n 65. The table is taken directly from *Mindspace* and I have added in the heuristics, biases, and effects which might be at play in some of the shorthand categories of influence.

69 Thaler and Sunstein 2009, above, n 4, ch 5.

70 COBIT, above, n 23, 8. In this regard, we fall prey to the availability heuristic; that is, we assess the frequency of an event based on the ease of which examples can be brought to mind. ‘Incidents of alcohol abuse are easily recalled’ (Thaler and Sunstein 2009, above, n 4, 73).

71 RB Cialdini, ‘Crafting Normative Messages to Protect the Environment’ (2003) 12 Current Directions Psychol Sci 105. For an example of the power of norms on wearing seatbelts in vehicles see J Linkenbach and HW Perkins, Most of Us Wear Seatbelts: The Process and Outcomes of a 3-year Statewide Adult Seatbelt Campaign in Montana. Presented at the National Conference on the Social Norms Model, Boston, MA, 17 July 2003 (see also <http://www.mostofus.org/project-gallery/traffic-safety/2010/most-of-us%C2%AE-wear-seatbelts-campaign-2002-2003> last accessed 2 July 2013).

72 K Yeung, ‘Nudge as Fudge’ (2012) 75 MLR 122, 125.

73 Thaler and Sunstein 2009, above, n 4, 73–4. See also JW Linkenbach, ‘The Montana Model: Development and Overview of a Seven-step Process for Implementing Macro-level Social Norms Campaigns’ in HW Perkins (ed), *The Social Norms Approach to Preventing School and College Age Substance Abuse* (Josseey-Bass, San Francisco 2003) 182–208; JW Linkenbach and HW Perkins, ‘Most of As Are Tobacco Free: An Eight-month Social Norms Campaign Reducing Youth Initiation of Smoking in Montana’ in HW Perkins (ed), *The Social Norms Approach to Preventing School and College Age Substance Abuse* (Josseey-Bass, San Francisco 2003) 224–34.

74 COBIT, above, n 7, 9.

75 Ibid at 19–22.

76 The much cited example of this is the ‘piano stairs’ where steps on a stairs were turned into piano keys which played music when stepped on. See <www.rolighetsteorin.se>, <www.youtube.com/watch?v=ivg56TX9kWI>, and <www.thefuntheory.com> last accessed 2 July 2013. Another non-health-related example of using novelty and fun to alter behaviour is with recycling. Turning a glass recycling bin into a ‘bottle bank arcade game’ where points could be scored for depositing glass bottles increased the number being recycled (in comparison to a nearby ordinary bottle bank, see <http://www.youtube.com/watch?feature=player_embedded&v=zSiHjMU-MUo> and <www.thefuntheory.com> last accessed 2 July 2013.

77 See Thaler and Sunstein 2009, above, n 4, 1–4 and Sunstein and Thaler, above, n 3, 1164–6. I follow Luc Bovens by saying that this is a paradigm case of nudging. See Bovens, above, n 10, 207.

78 Rozin and others, above, n 29.

79 Ibid at 329.

80 Ibid at 325–9.

81 Amir and Lobel, above, n 56, 2120.

82 Kahneman, Knetsch, and Thaler, above, n 61, 197–9.

83 Acute Respiratory Distress Syndrome Network, ‘Ventilation with Lower Tidal Volumes as Compared with Traditional Tidal Volumes for Acute Lung Injury and the Acute Respiratory Distress Syndrome’ (2000) 342 NEJM 1301.

84 For a discussion of this in the context of the Danish breast-screening programme, see T Ploug, S Holm, and J Brodersen, ‘To Nudge or Not to Nudge: Cancer Screening Programmes & the Limits of Libertarian Paternalism’ (2012) 66 J Epidemiol Commun H 1193.

85 Sunstein and Thaler, above, n 3, 1164. See also Hausman and Welch, above, n 10, 132.

86 Ibid.

87 Amir and Lobel, above, n 56, 2117–27. See also G Mitchell, ‘Libertarian Paternalism is an Oxymoron’ (2005) 99 NWULR 1245–77.

88 Yeung, above, n 72, 135.

89 Ibid at 136. See also Bovens, above, n 10.

90 See White, above, n 10, 110–3, Bovens, above, n 10, 218, and Yeung, above, n 72, 145–6.

91 White, above, n 10.

92 Ibid*.* See also Hausman and Welch, above, n 10, 124.

93 Thaler and Sunstein 2009, above, n 4, 6.

94 Hausman and Welch, above, n 10, 125.

95 The autonomy worry is something which has loomed large in the writing of both critiques and supporters of nudging thus far. See Hausman and Welch, above, n 10, 128–30; Yeung, above, n 72, 135–38; White, above, n 10, 105–7; Bovens, above, n 10.

96 Hausman and Welch, above, n 10, 130.

97 Feinberg, above, n 11, 28. Note that Feinberg talks of four different, albeit related, senses of autonomy: capacity, actual self-government, ideal of character, and sovereign authority.

98 G Dworkin, *The Theory and Practice of Autonomy* (Cambridge University Press, Cambridge 1988, reprinted 1991) 12–3.

99 I Berlin, ‘Two Concepts of Liberty’ in H Hardy (ed), *Liberty, Incorporating ‘Four Essays on Liberty’* (Oxford University Press, Oxford 1969) 166, 178.

100 Yeung and Morgan, above, n 42, 3–4.

101 It is not necessarily behaviour alteration per se that is the core of the objections, but the manner in which it is being done.

102 Berlin, above, n 99, 178.

103 See JS Mill, *On Liberty and Other Essays*, Edited with an Introduction and Notes by J Gray (Oxford University Press, New York 1991); Berlin, above, n 99; Feinberg, above, n 11, ch 18.

104 Yeung, above, n 72, 135.

105 Bovens, above, n 10, 209–10.

106 D Archard, ‘Informed Consent: Autonomy and Self-ownership’ (2008) 25 J App Phil 19, 21 [emphasis in original].

107 See L Haworth, *Autonomy: An Essay in Philosophical Psychology and Ethics* (Yale University Press, New Haven 1986) 39.

108 For the idea of bounded rationality see H Simon, *Sciences of the Artificial* (3rd edn MIT Press 1999).

109 Hausman and Welch, above, n 10, 135 [emphasis added].

110 Ibid.

111 Ibid.

112 White, above, n 10, 111.

113 Granted it is often difficult to determine what ‘intentions’ (or indeed if there are any) lie behind certain policies. See PG Hansen and AM Jespersen, ‘Nudge and the Manipulation of Choice: A Framework for the Responsible Use of the Nudge Approach to Behaviour Change in Public Policy’ (2013) 1 EJRR 3. Here the authors discuss the relevance of ‘intentionality (10–11) and argue that this imposes responsibilities upon policy-makers (23–27).

114 We might also conceive of these on a spectrum of micro to macro; for example, the alterations to the salad bar described earlier might be thought of as designed at the level of the cafeteria, but is more akin to a random aspect of choice architecture when taken in the wider context of a person's life or the general structure of society.

115 White, above, n 10, 111.

116 In talking of interests and preferences, I am mindful of Sunstein and Thaler's claim that ‘contextual influences render the very meaning of the term “preferences” unclear’ (‘Libertarian Paternalism’, 1161). However, for the purposes of this article, I assume that it is possible to attribute persons with such things as ‘true’ preferences and interests.

117 Yeung, above, n 72, 129 [emphasis added].

118 Hausman and Welch, above, n 10, 134.

119 A Carter and W Hall, ‘Avoiding Selective Ethical Objections to Nudges’ (2012) 12 Am J Bioethics 12, 13. I say behavioural science orientated efforts here because, as already noted earlier, in some sense all government policy is an attempt to influence behaviour one way or the other.

120 Thank you to John Coggon for helping to clarify my thoughts in this section.

121 Ibid [emphasis in original].

122 A Alemanno, ‘Nudging Smokers: The Behavioural Turn of Tobacco Risk Regulation’ (2012) 1 EJRR 32, 36. See also G Lowenstein, ‘Out of Control: Visceral Influences on Behavior’ (1996) 65 Organ Behave Hum Dec 272; JD Hanson and DA Kysar, ‘Taking Behavioralism Seriously: Some Evidence of Market Manipulation’ (1999) 112 Harvard Law Rev 1420. In 2012, the UK government conducted a consultation on removing all branding from cigarette packets. See <http://consultations.dh.gov.uk/tobacco/standar dised-packaging-of-tobacco-products/consult_view> last accessed 16 May 2013. See also a systematic review that was commissioned by the government on the impact of using plain packaging <http://phrc.lshtm.ac.uk/papers/PHRC_006_Final_Report.pdf> last accessed 2 July 2013.

123 Carter and Hall, above n 119, 13.

124 Ibid.

125 There is, of course, an issue of legitimacy with strategies such as the responsibility deal in relation to alcohol outlined earlier in this article. Where the government uses corporate actors as intermediaries in order to execute their public policy measures, the public/private distinction becomes blurry. Questions need to be asked about the appropriateness of private actors essentially acting as state regulators. Thank you to Elen Stokes for this point.

126 More will be said on this in the concluding section.

127 Ibid.

128 White, above, n 10, 110.

129 See Kahneman, above, n 54, 41–2. For an overview article of some of the studies and research in the area, see RF Baumeister and KD Vohs, Self-Regulation, Ego Depletion, and Motivation (2007) 1 Soc Personal Psychol Compass 115.

130 Kahneman, above, n 54, at 42. Of course while foods themselves cannot really be said to be ‘virtuous’, ‘bad’, or the like, our behaviour over time with respect to certain types of foods might mean that they are better or worse for our health and well-being.

131 Ibid.

132 Ibid.

133 Ibid at 41.

134 Hausman and Welch, above, n 10, 134.

135 A more complex worry, which requires further exploration, might be that individuals might fare worse when taken out of the contexts which are supportive or facilitative of certain aspects of their decision-making; that is, they will not learn to resist influences in more hostile choice environments. Thank you to Kalle Grill for this point. For a discussion of revealed versus normative (‘true’) preferences and strategies which might favour one over the other see J Beshears and others, ‘How Are Preferences Revealed?’ (2008) 92 J Pub Econ 1787.

136 Amir and Lobel, above, n 56, 2121.

137 White, above, n 10, 105.

138 I say ‘potentially’ because this is a claim that would need more work to be done in support of it since it is up for debate that these actually constitute legitimate ends of government. I make the assumption here that, to the extent that the government has obligations to its citizens in respect of health, these seem to be good candidates for such ends.

139 RH Frank, ‘Review of *Nudge*’ (2008) 119 Ethics 202, 205.

140 M Verweij and M van den Hoven, ‘Nudges in Public Health: Paternalism Is Paramount’ (2012) 12 AJOB 16, 16. For a similar point see Amir and Lobel, above, n 56, 2124.

141 Schlag, above, n 16, 916. On a related note, see also Bovens who says that ‘[a]dvertisement for products that do not increase welfare may use all kinds of Nudge style techniques and the government may be fighting a losing battle against, say, obesity, if it cannot access the same arsenal of techniques’ (above, n 10, 218).

142 Burgess, above, n 22, 11 [emphasis added].

143 R Korobkin, ‘What Comes After Victory for Behavioral Law & Economics’ (2011) U Ill L Rev 1653, 1655.

144 Yeung, above, n 72, 124, 146.

145 Amir and Lobel, above, n 56, 2110–4.

146 E Selinger and KP Whyte, ‘Nudging Cannot Solve Complex Policy Problems’ (2012) 1 EJRR 26. See also House of Lords' Report, above, n 5; T Marteau and others, ‘Judging Nudging: Can Nudging Improve Population Health?’ (2011) 342 BMJ 263, 265. Although as John and Richardson point out there is also a lack of evidence about the effectiveness of other approaches (above, n 37, 26).

147 Thank you to Elen Stokes for her thoughts on this point.

148 Marmot Review Working Committee 3, Cross-cutting Sub-group Report, *Learning Lessons from the Past: Shaping a Different Future*, November 2009 (available at <http://www.instituteofhealthequity.org/projects/the-marmot-review-working-committee-3-report> last accessed 2 July 2013) 3.

149 See this recommendation in the Marmot Review, above, n 34. Although note Sreenivasan's disquiet regarding the causal link between health and social factors. He maintains that ‘[i]t is not clear that our existing understanding of the causal pathways between socially controllable factors and specific mortality risks is sufficiently well-developed to underwrite concrete policy proposals.’ We, therefore, need to ensure empirical robustness whatever our posited causal mechanism and policy solution.

150 Amir and Lobel, above, n 56, 2116.

151 See the recent COBIT report on the potential for using randomised controlled trials to test public policy initiatives. L Haynes and others, *Test, Learn, Adapt: Developing Public Policy with Randomised Controlled Trials* (Cabinet Office behavioural Insights Team 2012) (available at <http://www.cabinetoffice.gov.uk/sites/default/files/resources/TLA-1906126.pdf> last accessed 2 July 2013). Although as Coggon notes, evidence of effectiveness (or lack of it) in itself does not speak to the political legitimacy of nudging as a measure (Coggon, above, n 12, 256–7).

